# Insights into how development and life-history dynamics shape the evolution of venom

**DOI:** 10.1186/s13227-020-00171-w

**Published:** 2021-01-07

**Authors:** Joachim M. Surm, Yehu Moran

**Affiliations:** Department of Ecology, Evolution and Behavior, Alexander Silberman Institute of Life Sciences, The Hebrew University of Jerusalem, 9190401 Jerusalem, Israel

**Keywords:** Toxins, Spatiotemporal gene expression, Predation, Defense, Convergent evolution, Complex trait, Sexual dimorphism, Ontogeny

## Abstract

Venomous animals are a striking example of the convergent evolution of a complex trait. These animals have independently evolved an apparatus that synthesizes, stores, and secretes a mixture of toxic compounds to the target animal through the infliction of a wound. Among these distantly related animals, some can modulate and compartmentalize functionally distinct venoms related to predation and defense. A process to separate distinct venoms can occur within and across complex life cycles as well as more streamlined ontogenies, depending on their life-history requirements. Moreover, the morphological and cellular complexity of the venom apparatus likely facilitates the functional diversity of venom deployed within a given life stage. Intersexual variation of venoms has also evolved further contributing to the massive diversity of toxic compounds characterized in these animals. These changes in the biochemical phenotype of venom can directly affect the fitness of these animals, having important implications in their diet, behavior, and mating biology. In this review, we explore the current literature that is unraveling the temporal dynamics of the venom system that are required by these animals to meet their ecological functions. These recent findings have important consequences in understanding the evolution and development of a convergent complex trait and its organismal and ecological implications.

## Introduction

Venom has fascinated humanity for thousands of years as fragile, small, and physically weak animals can deploy toxic cocktails that threaten the life of much larger and powerful animals, including humans [1]. This toxic mixture of chemicals is produced by one animal and is introduced via a wound infliction into another animal, causing upon its introduction an array of physiological and biochemical imbalances in the attacked animal [2]. The dominant proportion of these compounds found in venom is often proteinaceous and encoded by the animal’s genome [3]. The genes encoding toxin peptides are incredibly diverse, with many even having a restricted distribution to a specific lineage [4, 5]. Taken together with evidence that toxins regularly undergo rapid evolution under the strong influence of natural selection, venom has emerged as a model for extreme evolutionary trends and novelty [3, 6–8].

Changes in toxin expression may also combine to generate distinct venom profiles localized to specific tissues, life stages, or sexes that are essential for ecological functions, such as prey capture and defense [8–10]. The venom system itself is dynamic across the life history of venomous animals, undergoing both morphological and biochemical transitions that coincide with shifts in biotic interactions. Additional levels of complexity are also present with multiple different venom profiles capable of being produced within a given life stage.

**Fig. 1:**
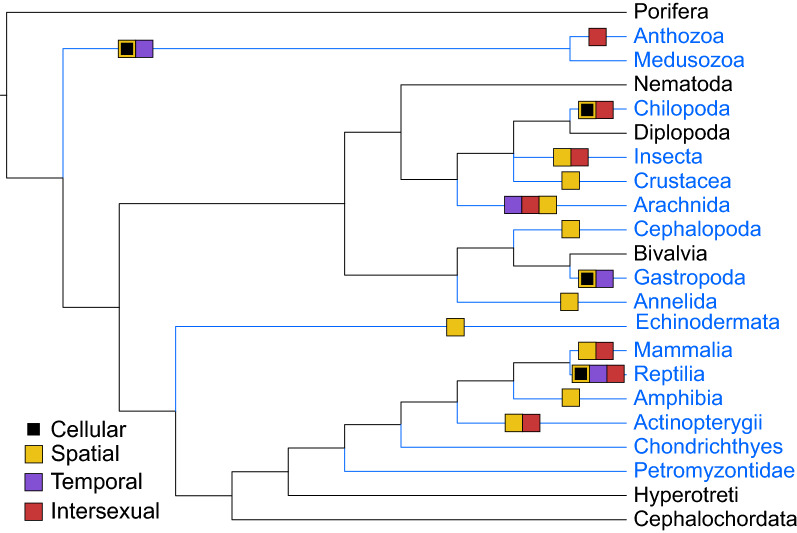
The convergent evolution for the separation of venom composition in animals. Lineages with known venomous taxa are depicted in blue. Boxes on branches highlight the evolution of venomous lineages that exhibit venom heterogeneity among morphological (spatial) and cellular structures, life history (temporal), and sexes (intersexual). The spatial separation of venom is predicted according to Table 2 provided in Schendel et al. [10], given evidence of animals that have a morphologically complex venom apparatus and putative multifunctional toxins profiles

Recently reviewed in Schendel et al. [10], the venom apparatus can contribute to the dynamic nature of venom deployed by animals through a process of modulation and compartmentalization of toxin expression. This process relies on morphological complexity that allows for the separation of venom among anatomically distinct venom glands, cellular spatial heterogeneity within a venom gland, or even being distributed throughout an organism through the decentralization of the entire venom system [10]. In concert, evidence of venom variation among males and females has also been reported, highlighting that the developmental processes related to sex determination and differentiation contribute to generating an animal’s venom phenotype. Strikingly, this process for the variation of the venom system spatially, temporally, or intersexually has independently evolved multiple times among distantly related animals (Fig. 1).

These venom system dynamics are an important and novel link between venom, evolution, and development. Here we review evidence of the process involved in developing the venom system and explore evidence for the spatial, temporal, and intersexual variation in toxin expression. Further, we propose that the study of developmental aspects of venom systems and their evolution can now advance by linking to the discipline of evolutionary developmental biology (evo-devo).

## Developmental dynamics of the venom system

The generation of a novel venom system requires substantial innovations, at the very minimum it requires the recruitment and evolution of cells that will produce toxins and a mechanism to inflict wounds and deliver venom via these wounds. Such evolutionary innovations at the cellular and morphological levels would always require vast changes at the molecular and genetic levels to enable them. Here we will review evidence of changes to the venom system both morphologically and biochemically across ontogeny.

### Venom apparatus development

Snakes are among the most studied venomous animals, largely due to the significant adverse effect of their bites on human health (reviewed in Gutiérrez et al. [11]). While venom composition has been the subject of the majority of these studies [12], research investigating the development of their venom apparatus is attracting considerable attention from evolutionary and developmental biologists [8, 10]. This system typically consists of a gland loaded with venom and delivered using specialized fangs [13–15]. These fangs are often located on the maxilla and are distinct from the other tooth-bearing bones [16]. Broadly, the snake fang phenotype is highly heterogeneous, differing in its location in the jaw as well as other various characteristics, including tooth morphology that can be either grooved, hollow, or tubular [14, 17–19].

Significant insights into the origin and evolution of the fang phenotype were revealed using developmental genetics. Specifically, Vonk et al. [14] performed in situ hybridization of the *sonic hedgehog* (*SHH*) gene on serial sections of snake embryos to reconstruct three-dimensionally the development of snake fangs. The findings from this work revealed that front and rear fangs share striking similarities in their morphogenesis, both of which develop from the posterior end of the upper jaw. During front-fang development, ontogenetic allometry occurs which displaces the fang from its posterior developmental origin, transitioning to the anterior position in adults [14]. In contrast, rear-fanged snakes retain their posterior positioning which develops from an independent posterior dental lamina [14]. This work, among others, provided support that front and rear fangs are homologous and likely evolved from a rear-fanged ancestor [14, 17–19]. The subsequent radiation of snakes led to multiple independent gains and losses of various fang phenotypes [19]. Recent work has highlighted that the evolution of the rear-fang phenotype in snakes is highly dynamic, exhibiting extreme heterogeneity compared to the front-fang phenotype which appears to be much more stable [19]. The acquisition and evolution of venom in snakes have likely shaped fang morphology, specifically with fangs from colubrid snakes transitioning more anteriorly [19].

To date, our understanding of the development and evolution of the venom gland has remained largely unresolved in most venomous species. A recent review by Zancolli and Casewell [8] highlights that venomous lineages share a common trait in which they possess specialized epithelial cells that synthesize, store, and eventually secrete venom components. The collective organization of these cells can form a conspicuous venom gland that is found in most venomous animals [8]. In snakes, the venom gland forms during development from oral tissue, suggesting it is derived from the salivary glands [15]. However, an alternative hypothesis has been suggested that the venom gland is derived from the pancreas [15]. Evidence for this is supported by the expression of a microRNA (miR-375) in the venom gland of the king cobra that is also found in the pancreas of other vertebrates [20]. However, further evidence is needed to confirm this hypothesis, such as the co-option of the pancreatic gene-regulatory network to the venom gland. To gather such insights, functional assays and genomic studies investigating the mechanisms related to the development of the venom system are needed. This requires novel techniques and technologies that until now were not accessible.

Organoids are a revolutionary new technique that has been developed to enable the recapitulation of essential features, tissues, and organs into 3D biological structures [21]. This requires defined growth factor conditions from adult stem cells (ASCs). Advancements in the use of serum-free medium containing R-spondin, the BMP (bone morphogenic protein) inhibitor Noggin, and EGF (epidermal growth factor) enabled the growth of mouse intestinal ASCs into an epithelial organoid [22]. Following this breakthrough, additional R-spondin-based protocols have been implemented to recapitulate both healthy and diseased mammalian epithelia, including growing mammalian salivary gland organoids [23, 24]. These insights led to researchers being able to recapitulate the snake venom gland as an organoid [25]. This was achieved by first dissociating snake venom glands and embedding them into basement membrane extract. The initial expansion of organoids was made possible by supplying a medium containing a “generic” mammalian organoid cocktail. Further expansion was induced using R-spondin, Noggin, EGF, the small molecule TGF (Transforming growth factor) beta inhibitor A83-01, PGE2 (Prostaglandin E2), and FGF10 (fibroblast growth factor 10). Strikingly, this “expansion medium” controls the same cellular signaling pathways that are required for mammalian epithelial organoids. This provides evidence that many of the same factors controlling the development of mammalian epithelium are also active in reptiles and were probably recruited as whole developmental modules into the venom system. Exploring whether these factors also control the development of other vertebrate venom glands remains to be tested. The development of the first venom gland organoid suggests that we are on the precipice of exciting breakthroughs in understanding the evolution and development of venom glands.

#### Temporal variability of toxin expression across ontogeny

The formation of the complete and functional venom apparatus allows for the utilization of venom to collectively function in ecological roles, such as predation and defense [26–29]. In some venomous animals, the formation of the venom system occurs at a juvenile stage that may have unique biotic interactions compared to adults. Here we will review evidence of venomous animals that have evolved the ability to express different toxins at the juvenile and adult life stage.

In multiple snake species, variations in venom composition have been associated with ontogeny [30]. This was first documented in detail by Mackessy in 1988 [31]. In this seminal work, the ontogenetic variation in venom composition was examined in rattlesnakes of various lengths [31]. In both *Crotalus helleri* and *Crotalus oreganus*, increased protease activity is positively correlated with size, and toxicity is more pronounced in juveniles [31]. The separation of venom pharmacology among ontogenetic changes may have evolved due to changes in diet requirements. This was supported by analysis of the gut contents of museum specimens of the *Crotalus* species, with lizards contributing to a major proportion of the diet in juvenile snakes, whereas mammals are the primary diet of adults [31]. The resulting juvenile rattlesnake venom composition is one of high toxicity and with low protease activity that efficiently targets lizards and small rodents. It is proposed that adult snakes that target larger mammals require protease activity to digest their prey effectively [31].

Similar patterns of ontogenetic variation have also been observed in other rattlesnake species. For example, the transcriptomes from *Crotalus adamanteus* adult and juvenile venom glands were sequenced from five populations, revealing that 12 from 59 toxin transcripts exhibit significant differential expression across ontogeny [32]. From these 12 differentially expressed toxins, three and nine toxins were upregulated in juveniles and adults, respectively. While similar total levels of snake venom metalloproteinases were expressed in adults and juveniles, paralog-specific expression was observed to be restricted to ontogenetic stages [32]. In adults, specific paralogs of phospholipases A_2_ were upregulated, along with Bradykinin-potentiating and C-type natriuretic peptides, nerve growth factor, and snake venom serine proteinases. Consistent with Mackessy [31], juvenile venom was also identified to be more toxic to small rodents [31, 32]. This provides evidence that the pharmacological plasticity of venom may be driven by temporal changes in the expression of toxin-encoding genes. Further evidence from species in the *Crotalus* genus report that similar patterns are also observed. For example, the venom proteome of 6-week-old *Crotalus simus* is predominantly composed of neurotoxins, while the major adult venom components are snake venom metalloproteinases [28]. These ontogenetic differences in the production of toxins generating phenotypically divergent profiles result in adult and newborn venom being hemorrhagic and neurotoxic, respectively.

Conspicuous changes in venom composition are also observed across ontogeny in other snake species, including pit vipers (genus *Bothrops*) [33, 34], as well as brown snakes (genus *Pseudonaja*) [35, 36]. For example, *Bothrops* venoms showed differential toxicity and pharmacology in newborn and juvenile with higher lethality in mice compared to adults [33]. This is likely due to newborns and juveniles having increased hemorrhagic, edema-forming, and coagulant activities. In *Bothrops jararaca*, the newborn venom is highly lethal to chicks (*Gallus gallus*), whereas the adult venom has a slightly higher lethal activity in mice [34]. Ontogenetic changes in the venom composition among species of brown snakes (*Pseudonaja)* have also been reported, revealing shifts in the functional activity of the venom profile during the transition from juveniles to adults [35, 36]. For example, Cipriani et al. [35] revealed that many species of *Pseudonaja* transitioned from expressing non-coagulopathic venom in juveniles to coagulopathic venom as adults. Again, these ontogenetic shifts in venom activity correlate with dietary preference dynamics across life history, with most juvenile brown snakes preferring reptiles as prey and transitioning to become more generalized predators in adults [35, 36]. Differences between young and adult snake venom profiles can also be found in distantly related snake species from the Colubridae family. For example, in the rear-fanged snake *Boiga irregularis*, venom underwent an ontogenetic shift in enzyme activities and toxicity, with younger snakes producing more toxic venoms with lower protease activities [37].

The organoid of the Cape coral snake (*Aspidelaps lubricus cowlesi*) also revealed interesting insights into the temporal expression of toxins [25]. As the organoid is exposed to different cocktails of media, it first undergoes an expansion phase, then differentiates to generate mature and functional cell types [25]. While all toxins increased their expression across these phases with different mediums, the CRISP (cysteine-rich secretory protein) toxin underwent an inverse pattern and its expression levels dropped when transitioning from the expansion to the differentiation phases. These results hint toward potential temporal venom dynamics previously unreported, with the CRISP toxin potentially being expressed during early life stages.

Evidence of venom composition changes between juveniles and adults is also reported in the tarantula, *Phlogius crassipes* [38]. The venom profiles from four ontogenetic stages of this species were examined according to cephalothorax length using gel electrophoresis and mass spectrometry [38]. This revealed that some potential toxins are expressed only in a specific ontogenetic stage; however, the function of the toxins remains to be characterized. Whether this is unique to tarantulas among spiders remains to be tested. In concert, ontogenetic differences in venom composition have also been reported in the Brazilian spider (*Phoneutria nigriventer*), with the venom profile shifting to become predominantly composed of low-molecular weight proteins in adults [39]. This shift in venom composition likely contributes to adult venom having increased lethality in mice [39]. Evidence of ontogenetic differences in the expression of toxins in animals has also been reported between dramatically different life stages, such as gametes, developing larvae, and adults, in addition to more nuanced life stages, i.e., juveniles and adults.

### Venom apparatus development across a complex life cycle

Venomous animals that undergo a complex life cycle rely on the coordination of the venom system with transitions in their life stages. Dynamic morphological and biochemical shifts must coincide with changes to their ecological requirements, such as predator–prey interactions. To date, this phenomenon has been explored in detail in *Nematostella vectensis*, which can complete its full life cycle in the lab [40, 41].

In members of the phylum Cnidaria (corals, hydroids, sea anemones, and jellyfish), there is no centralized venom gland; instead, various types of cnidocytes (“stinging cells,” a synapomorphy that typifies this phylum) have evolved. Cnidocytes harbor the cnidocyst, arguably the most morphologically complex organelle known to date, which is a harpoon-like structure that discharges at an incredible speed and force, punctures the cuticle and/or epidermis of the stung animal, and delivers venom [42–44]. Numerous types of cnidocysts have been characterized, some of which have a restricted distribution among specific cnidarian lineages. For example, spirocysts are unique to Anthozoa (corals and sea anemones) and are used to entangle the target animals using thread-like organelles. Contrastingly, nematocysts are organelles that serve as a microinjector to deliver the venom and have a much broader distribution in Cnidaria (reviewed Kass-Simon and Scappaticci [45]). This suggests that nematocysts are likely the ancestral cnidocyst [42].

Studies on the model cnidarians, *N. vectensis* (Anthozoa), *Hydra magnipapillata,* and *Hydra vulgaris* (Hydrozoa), have provided unparalleled insights into the development and functions of the venom apparatus components. For example, in *Hydra*, the maturation of cnidocytes occurs following their differentiation from interstitial cells (i-cells; for reviews see [46, 47]). These i-cells are hydrozoan-specific progenitor cells found throughout the mid-gastric region of the ectoderm [46–48]. The specialized organelle, the cnidocyst, develops within a post-Golgi vesicle during differentiation from i-cell to cnidocyte [46, 47]. The cnidocyst compromises multiple structural proteins that generate the tubule, harpoon, and capsule wall [42, 47, 49, 50]. To date, many of these proteins are cnidarian specific, such as minicollagens and nematogalectins, and are regulated through posttranscriptional and posttranslational modifications, such as alternative splicing and preprotein cleavage, respectively [42, 47, 49, 50].

Additionally, recent studies have been revealing insights into the development of the three different cnidocyte cell types characterized in *N. vectensis*. These include two types of nematocytes (basitrichous isorhizas and microbasic p-mastigophores) and spirocytes [51]. The distribution and density of cnidocytes in *N. vectensis* vary across tissue and development, for example, basitrichous isorhizas are found in high density as early as the planula stage, whereas spirocytes can be found predominantly in tentacles after the primary polyp stage [51]. The development of cnidocytes in *N. vectensis* is driven by transcription factors, such as *SoxB2* which is expressed in a population of progenitor cells that can give rise to both neurons and cnidocytes [52], among others [53–55]. Interestingly, the homologous bilaterian SoxB genes are involved in neurogenesis as well [56], indicating that this role is conserved for hundreds of millions of years. Further, the existence of a common progenitor cell of neurons and cnidocytes in multiple cnidarians as well as the recent finding that nematocyte neurotoxins can be recruited from neurons in *N. vectensis* and possibly other cnidarians [57] support the notion that cnidocytes might be highly derived neurons [58, 59].

Many other venomous invertebrates also develop across a complex life cycle, undergoing metamorphosis and transitioning from a larval form to a juvenile and eventually becoming an adult. To date, understanding how the venom apparatus develops during these transitions remains an open question. An example that explores this process was reported by culturing the feeding larvae of *Conus lividus* [60]. Subsequent serial histological sections were performed by dissecting the foregut during larval and metamorphic stages to trace the development of the venom gland [60]. These results provide support for the hypothesis of homology between the venom gland and the mid-esophageal gland of other gastropods. The development of the venom gland may also differ depending on whether the cone snail feeds at the larval stage. Results suggest that the venom gland of *Conus anemone*, which has a non-feeding larval stage, may develop through a different process that involves the out-pocketing of the ventral glandular region of the foregut [61]. While these different processes occur in generating the venom glands of *C. anemone* and *C. lividus*, they both share similarities in their formation and accumulation of secretion granules within the presumptive venom gland prior to larval metamorphosis [41, 42]. This suggests that these cone snails begin loading their venom gland before transitioning to juvenile snails. Whether this can be used and injected remains to be elucidated. While histological and morphological assays are providing key insights into the development of the convoluted venom apparatus in cone snails, the molecular pathways that control this process have yet to be characterized.

In other invertebrates, the completion of the venom apparatus coincides with their feeding requirements. For example, the staining of *Strigamia maritima* embryo using DAPI revealed that the venom apparatus is likely formed during early postembryonic development [62]. This is consistent with evidence that in the early stages of post-hatching, *S*. *maritima* is incapable of feeding using their forcipules, which are derived from a pair of walking legs. This is also similar for *Scolopendra subspinipes mutilans* [62, 63] in which the venom gland is first observed eight days after the molt and during the transition from the postembryonic II to the fetus stage [64]. As the centipede continues to transition from the fetus stage, the pre-adult centipede develops well-formed forcipules (which are heavily sclerotized and fully functional) and a complete venom duct. At this stage, the centipede is capable of feeding with only the venom duct eventually becoming detached from the endocuticle of the exoskeleton.

Evidence of a developed venom apparatus at the larval stage of spiders has also been reported. In *Phoneutria nigriventer*, scanning electron microscopy revealed that at the larval stage that precedes the spider’s eclosion from the cocoon, the venom apparatus has developed a bilaterally symmetrical pair of ducts, chelicerae, and venom glands that display their characteristic shape and are surrounded by a layer of muscle [65]. This suggests that the venom apparatus has completely formed at this early life stage. While this precedes the animal’s ability to capture prey, the venom system may play a role in defense during this early life stage. As predation becomes necessary, the venom glands of *P*. *nigriventer* begin to transition internally to the prosoma of the adult [65]. Whether this transition helps mediate the spider’s ability to use venom for prey capture and feeding remains to be tested.

#### Temporal variation of toxin expression across complex life cycles

**Fig. 2:**
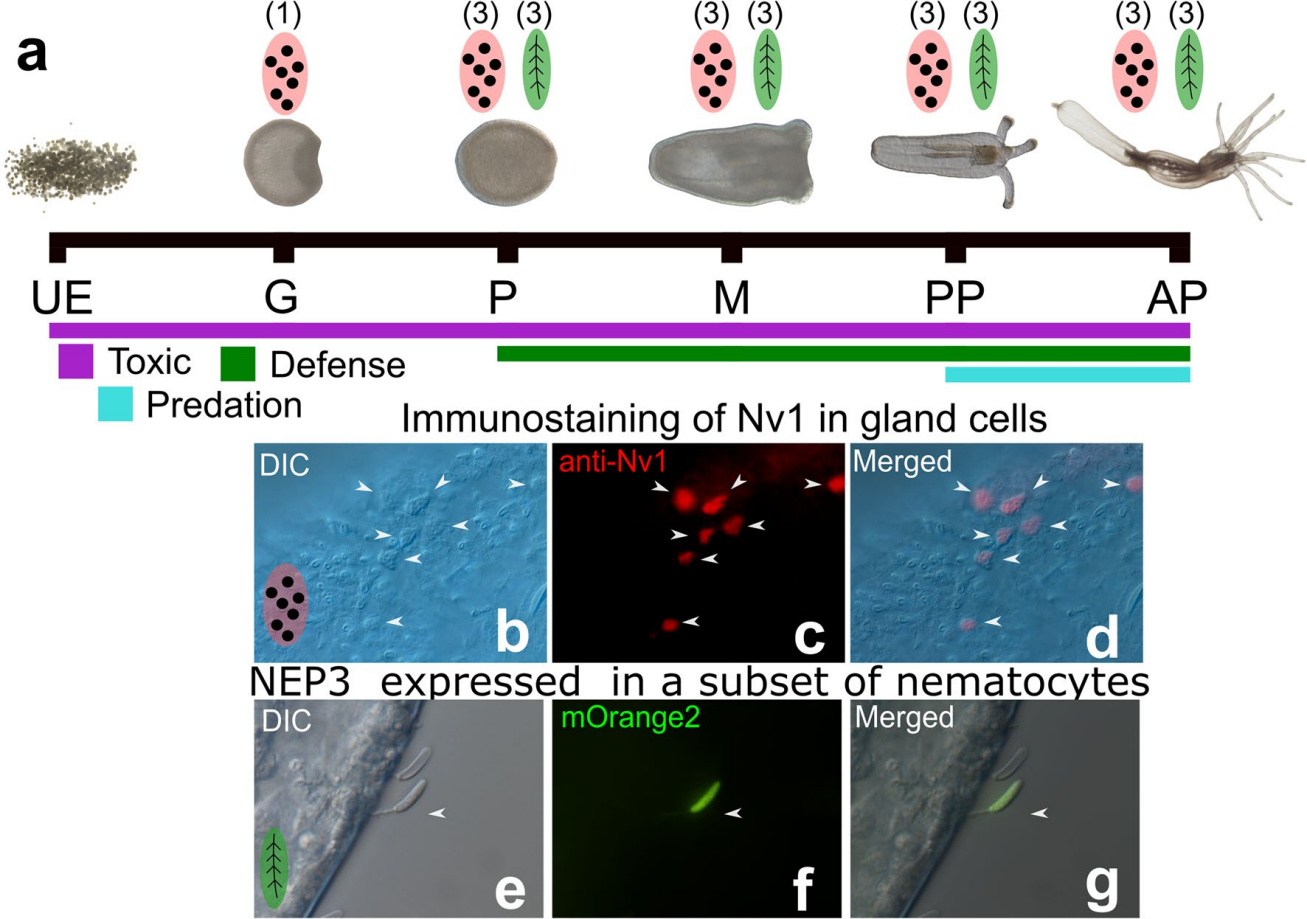
Development of the venom system in the model sea anemone *Nematostella vectensis.* **a** The life cycle and timeline of *N. vectensis*, with a conservative estimate of the occurrence and diversity of venom-secreting cells. The shifting roles of venom across the development of *N. vectensis*, from being toxic (purple) to having additional defensive (dark green) and predatory (cyan) functions. Schematic representing the minimum number of types of gland cells (red) and nematocytes (green). **b–d** Immunostaining Nv1 localized in ectodermal gland cells of *N. vectensis* polyp [69]. **e–g** Nematocytes from transgenic polyp expressing mOrange2 under control of NEP3 promoter and embedded in the cuticle of *Artemia salina*. Only one nematocyte in the picture is mOrange2 positive [70]. UE unfertilized eggs, G gastrula, P planula, M metamorphosis, PP primary polyp, AP Adult polyp

*N. vectensis* was established in the last two decades as an important lab model in the field of evolutionary developmental biology [40, 52, 66, 67]. During the life cycle of *N. vectensis* (Fig. 2), both males and females release gametes to the water via spawning [68]. Following fertilization, the zygote cleavage begins forming a blastula, and subsequent gastrulation is completed in less than 24 h post-fertilization (hpf). A planula larva emerges from the egg package 48–72 hpf and starts swimming in the water. 6 to 7 days after fertilization, the planula settles in a soft substrate and starts to metamorphose into a primary polyp and sexual maturation takes about 4 months under lab condition.

Toxins can be delivered using two different cell types: nematocytes which develop as early as 48 hpf in the swimming planula [58], and gland cells loaded with venom components and found even earlier at the gastrula stage (Fig. 2a). At least four different types of gland cells have been identified across the life history of *N*. *vectensis*, from as early as the gastrula stage [70]. This diversity of gland cells is supported by recent single-cell RNA sequencing (scRNA-seq) revealing that multiple gland cell populations express different toxins at different developmental stages [71]. Temporal dynamics in toxin expression have also been investigated using experimental approaches for a few key toxin gene families [70, 72–74].

Strikingly, Nv1 is the major venom component of the adult polyp’s venom profile (Fig. 2b–d) yet absent in the larval stages [69, 73, 75]. This toxin is produced in massive amounts which is likely a consequence of the highly conserved copies found in tandem in the genome, with more than 10 copies identified in the genome [70, 75]. The abundance of Nv1 distinctly at the polyp stage is even more striking, given that the multiple *Nv1* loci are transcribed at all developmental stages of *N. vectensis*; however, proper splicing of these transcripts is restricted to the polyp stage [73]. This is achieved through intron retention in the early life stages, a posttranscriptional regulatory mechanism in which functional Nv1 synthesis is restricted after the polyp stage and absent from the embryo and planula stages [73]. The production of Nv1 coincides with the requirement to capture prey, while venom produced in earlier life stages are likely specialized for defensive purposes, as the sea anemone does not feed before the primary polyp stage. The specialization of venom profiles has been attributed, at least partially, to the molecular mechanism of gene duplication, which has resulted in the diversification of toxins with divergent temporal expression and target specificity [76].

The recent characterization of *Nv1* paralogs has revealed a pattern of functional specialization divergent from other members of this gene family [76]. Specifically, *Nv4* and *Nv5* are expressed in early life stages, confirmed both quantitatively, (nCounter and LC–MS/MS) and qualitatively (transgenesis and immunostaining). At the protein level, Nv4 and Nv5 have specialized to be lethal to zebrafish larvae but harmless to arthropods, whereas Nv1 shows highly lethal against insects [73]. This pattern is supported in ecological studies in which natural fish predators avoid feeding on eggs and planulae of the anemone [70]. The evolution of the *Nv1* gene family has ultimately led to the adult-specific expression of *Nv1* coinciding with prey capture needs, and *Nv4* and *Nv5* expression in early life stages required for specialized defensive functions. Other toxin-encoding genes, such as *NvePtx1* and *NEP3*, have also been attributed to contributing to the resistance toward fish predators in the early life stages [70].

*NvePtx1*, a homolog of a known potassium channel blocking toxin, is expressed dynamically across the life cycle of *N. vectensis* [70, 74]. Both quantitative and qualitative approaches at both the RNA and protein levels revealed evidence of *NvePTx1* being expressed in gland cells early in development and subsequently downregulated following the transition to the polyp stage [70]. Nematocytes are also used to deliver venom during the early life stages of *N. vectensis*. Specifically, *NEP3* is expressed in nematocytes across development (Fig. 2e–g), starting as early as the planula stage [70]. While the spatial expression patterns of *NvePtx1* and *NEP3* are distinct, their expression in early life stages supports their utilization as defensive toxins [63]. These findings suggest that venomous animals with a complex life cycle that experience different ecological interactions may produce vastly different venoms in distinct life stages. Congruently, the temporal expression of toxins across complex life cycles has also been reported in multiple diverse taxa and will be reviewed here.

In the reef-building coral *Acropora millepora*, different members of the small cysteine-rich peptides (SCRiPs) neurotoxin gene family are upregulated at different developmental stages [77, 78]. Specifically, this family of neurotoxins exhibit dynamic temporal expression with *SCRiP3* being upregulated in the post-settlement stage, *SCRiP2* upregulated in the pre-settlement stage, and *SCRiP*-like upregulated in the adult [77, 78]. Furthermore, the expression of toxins in early life stages is present among other cnidarians [9]. This is evident with some pore-forming toxins expressed specifically in the embryo of *Hydra vulgaris* [79]. Evidence of ontogenetic differences in venom profiles has also been reported in cubozoans, with the Australian box jellyfish *Carukia barnesi* showing proteinaceous components of the venom extract having different molecular weights specific to immature and mature animals [80]. Furthermore, these differences are correlated to changes in diet preference in which young and adult medusae have invertebrate and vertebrate prey preference. The findings of toxins expressed early in development from distantly related species across Cnidaria suggests that this process is conserved among this venomous phylum. While this is likely conserved among cnidarians, similar patterns are observed in other venomous lineages, suggesting its convergent evolution.

Evidence of toxin expression across life stages has also been reported in cone snails [81]. In *Conus victoriae*, sequencing captured venom mRNA expression in embryos, revealing five novel O- and two α-conotoxin transcripts [81]. In addition to these novel toxins, the expression of a known adult toxin *Vc1.1* was also captured in the embryo. Functional assays revealed that the embryonic α-conotoxins have different neuronal nicotinic receptor targets, suggesting that they may have specialized functions or prey specificity [81]. Further systematic studies investigating the venom profile in early life stages is required to determine whether cone snail embryos and newly hatched juveniles may synthesize defense-specific venom essential to deter predators as observed in *N*. *vectensis*. In addition to venomous individuals that undergo dynamic toxin expression temporally, some also can generate distinct venom profiles spatially.

### Heterogeneity and compartmentalization of toxin production and its impact in venom profiles

A major insight into the separation of venom within a given life stage was reported in the scorpion, *Parabuthus transvaalicus* [82]. Upon stimulation, this scorpion initially secretes a prevenom cocktail that is transparent, with further stimulation resulting in a different secretion that is cloudy and white in color [82]. The components of these two distinct venom profiles vary in their combinations of salts and peptides. The prevenom is rich in potassium (K^+^) salts and contains some peptides that block voltage-gated K^+^ channels, resulting in local depolarization that ensures severe pain and toxicity in the target which is essential for defense purposes [82]. Venom secreted after the prevenom consists predominantly of peptides and proteins and is reported to have a less severe pain response, yet maintains a high potency and lethality to both mice and insects [82]. The separation of these two venoms suggests that the prevenom has evolved to be highly specialized for roles related to defense.

**Fig. 3:**
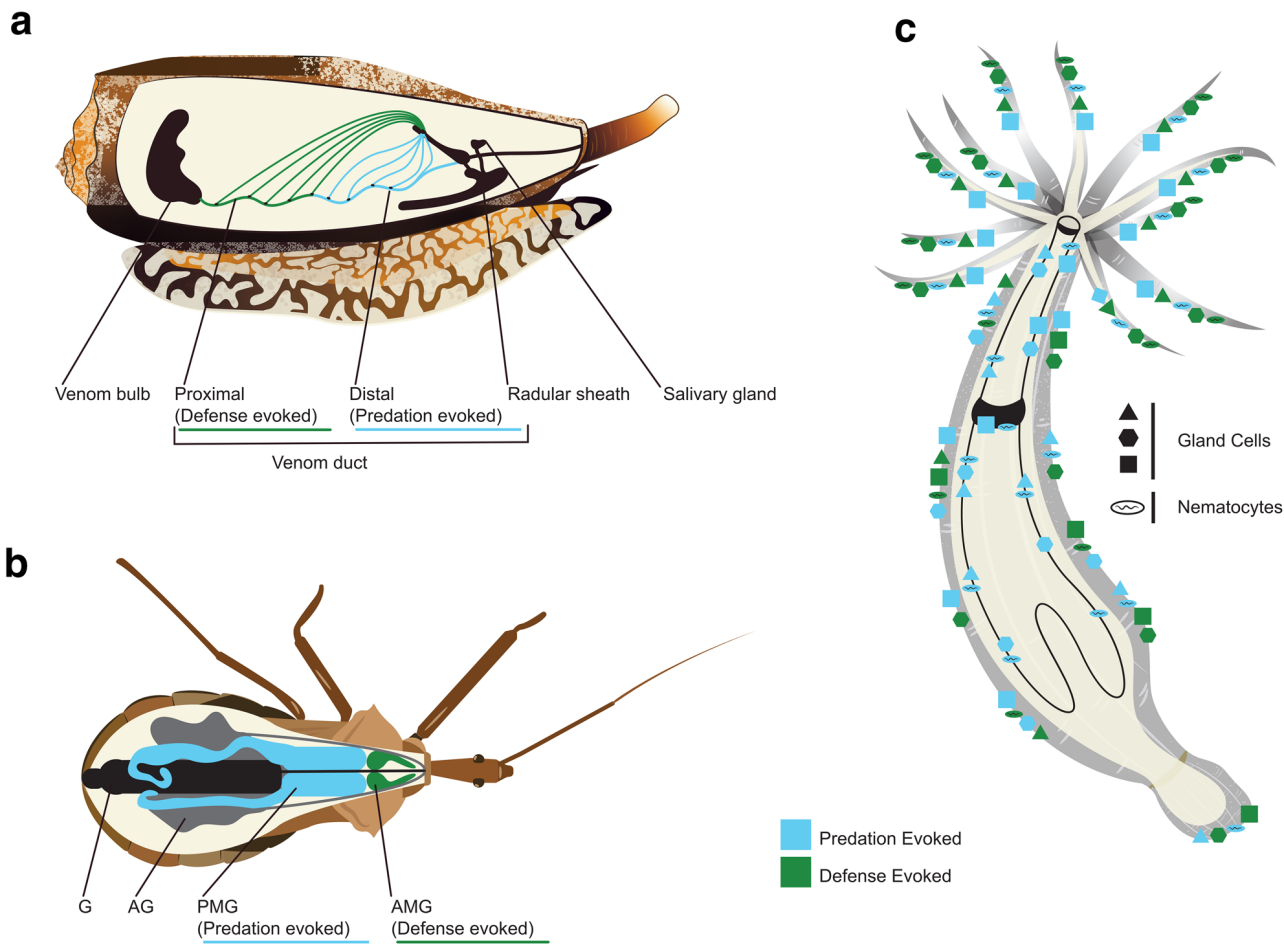
Spatial separation of functionally distinct venom in animals. **a** Cone snail. **b** Assassin bug; anterior main gland (AMG), posterior main gland (PMG), accessory gland (AG), and gut (G). **c** Sea anemone. Predation-evoked venom profile highlighted in cyan and defense-evoked venom highlighted in dark green

Multiple recent studies have reported a similar process for the separation of distinct venom profiles within a given life stage. Advancement in molecular techniques is revealing that this separation of venom is driven by the compartmentalization of toxin expression at the gross organ and tissue levels. For example, Dutertre et al. [83] revealed that cone snails can dynamically transition their venom composition in response to predatory or defensive stimuli (Fig. 3a). The defensive stimulus induces the production of high levels of paralytic toxins that efficiently block neuromuscular receptors in vertebrates, while the predatory stimulus induces the production of distinct venom with a composition enriched in predatory-specific toxins that are mostly inactive in vertebrates [83]. Evidence supports that this envenomation strategy is an ecologically important trait, with a defense-specific venom conserved among worm, mollusk, and fish-hunting cone snails [83–85]. These distinct venom profiles are produced through the regional heterogeneity in toxin expression. Specifically, the distal and proximal regions of the venom duct generate the predatory and defensive specific venoms, respectively [83].

The work by Post et al. [25] revealed a similar pattern of regional heterogeneity in toxin expression in the Cape coral snake, *Aspidelaps lubricus cowlesi*. This was achieved by dissecting its embryonic venom glands into proximal (located near the duct) and distal regions to generate region-specific organoids. Analysis of the toxin expression in region-specific organoids by scRNA-seq identified that C-type lectins are enriched in the proximal organoids, whereas distal organoids cells predominantly produced Kunitz-type protease inhibitors and three-finger toxins [25]. This is consistent with previous work that observed in the king cobra, C-type lectins are expressed in serous cells located in the proximal region of the accessory gland [20]. Whether this can be evoked through behavioral response or specific stimulus is beyond the limits of organoid research. Indeed, such insights would require work in a more organismal context. Recent advancements in the in situ mapping of toxins in the venom gland may allow for such potential insights.

Using a novel mass spectrometry imaging (MSI) method, Hamilton et al. [86] revealed the spatial distribution of venom activity across the snake venom gland. The venom glands of the brown forest cobra (*Naja subfulva*) [87] are rich in enzymatically active phospholipases A2 (PLA_2_) and sections exposed to phospholipid substrates produced high-resolution maps of phospholipase activity and specificity [86]. This novel method supports the heterogeneous distribution of venom components, including the PLA_2_, and three-finger toxins [86]. Intriguingly, the distribution of these venom components showed that their abundances are non-overlapping, in which the abundance of three-finger toxins in the posterior region of the gland has limited PLA_2_ activity [86].

The assassin bug (*Pristhesancus plagipennis*) is also capable of modulating the composition of their venom in a context-dependent manner, similar to that observed in the cone snail [88]. The assassin bug separates functionally distinct venom through the compartmentalization of toxin expression to different anatomical regions (Fig. 3b). This is evident from their complex venom system consisting of three distinct glands: the anterior main gland (AMG); posterior main gland (PMG); and accessory gland (AG). Using a combination of transcriptomics and proteomics, it was revealed that the AMG and PMG venom is generated following harassment and electrostimulation, respectively [88]. Specifically, the venom specific to the PMG potently paralyzes and kills prey insects, while the AMG-specific venom alternatively does not paralyze prey insects, further supporting its use for defense [88]. While the assassin bug uses distinct glands for the separation of venom, recent evidence from multiple different venomous lineages is revealing that this process may also occur at the cellular resolution.

#### Heterogeneity among venom-secreting cells

The dynamic expression of toxins among cells within a venom gland likely provides the cellular complexity required to generate functionally distinct venom profiles. Cellular compartmentalization of snake venom has only recently been elucidated following the development of venom gland organoids and scRNA-seq [25]. Previous work exploring the cellular diversity of the snake venom gland characterized four morphologically distinct cell types, with only one being the dominant cell type used to secrete venom [89]. Analysis using scRNA-seq revealed that specific toxins were strongly enriched in distinct populations of cells [25]. The evidence of cellular heterogeneity in toxin expression suggests that this organ may be more complex than previously characterized by morphology.

In cnidarians, the toxin delivery system is a complex of non-linked cells, involving multiple different cell types distributed heterogeneously throughout the organism [42, 45, 69, 90]. Cnidaria is the only phylum that shares a venomous ancestor, with members characterized by the presence of cnidocytes. These typifying cells are highly heterogeneous among cnidarians in their morphology and functions which range from prey capture, defense, as well as locomotion [45]. In *N. vectensis*, venom is also produced in gland cells as was initially revealed by the localization of Nv1 to these specialized ectodermal cells [69]. Recent evidence is reporting the compartmentalization of venom components among these highly specialized cells.

Nematocyte heterogeneity has also been observed among tissues in *Actinia tenebrosa*, with differences coinciding with changes in the expression of toxin-encoding genes. Morphological structures in *A*. *tenebrosa* with a high density of nematocytes include tentacles used in prey capture and defense, mesenteric filaments used in digestion and killing of prey, and acrorhagi used solely in intraspecific aggressive encounters [90, 91]. Nematocysts found in the acrorhagi consist predominately of holotrichs, whereas the tentacles and mesenteric filaments contain a higher proportion of basitrich nematocysts [92]. Acrorhagi are unique to the Actinioidea family and found to produce a distinct venom profile compared to tentacles and mesenteric filaments [91, 93]. Toxins with a restricted expression to the acrorhagi include *acrorhagin I* and *II* and are consistent with previous work that isolated these toxins from acrorhagi in the closely related species, *Actinia equina* [94]. This provides a correlation between the morphological type of a nematocyst and the expression of specific toxins. While this toxin is lethal against crustaceans [94], given the ecological function of the morphological structure, it is localized to, this toxin might also have specialized action against sea anemones, specifically those from the *Actinia* genus.

Differences in the expression of toxins among tissues and populations of cells have also been reported in other sea anemone species [95]. For example, in *Heteractis magnifica*, different members of a single pore-forming toxin family were found in single cells isolated from within and among different morphological structures (tentacle and body column, [95]). The cellular compartmentalization of toxins is also found in other cnidarians. For example, a study in *Hydra* revealed that two members of a single pore-forming toxin family are expressed in two morphologically distinct types of nematocytes and this trend was extended by a recent analysis of scRNA-seq data from this species [96, 97].

Tissue-specific variation of toxin-encoding genes and venom-secreting cells has been reported in multiple sea anemones species [5, 70, 98], providing evidence for the cellular and biochemical complexity of their venom system (Fig. 3c). This is clear in *N. vectensis,* with some members of the *NEP3* family showing patterns of expression localized to distinct cells and areas of the organism [70]. For example, members of the *NEP3* family (*NEP3*, *NEP3-like*, and *NEP4*) are expressed in nematocytes in the tentacles and outer body of *N. vectensis* [70]. In contrast, another *NEP3* paralog, *NEP8*, is absent from the tentacles and outer body wall but specifically expressed in pharyngeal nematocytes, suggesting it is involved specifically in the paralysis of swallowed prey [70]. This supports that different populations of nematocytes among tissues express distinct venom components.

Further findings into the dynamic expression of toxins in *N. vectensis* is providing evidence that the molecular diversity of nematocytes and gland cells exceeds their morphological diversity [70]. This is evident for the *NEP3* toxin which appears to be expressed in only a certain nematocyte subpopulation, even among neighboring nematocytes within the same tissue (Fig. 2e–g). Furthermore, potential differences among subpopulations of gland cells have also been reported in *N. vectensis*. Using in situ hybridization and transgenic animals (that express a fluorescent reporter under the promoter of the toxin-encoding gene *NvePTx1*), it was revealed that at least two distinct types of ectodermal gland cells are present in the *N. vectensis* planula, one large and elongated and another small and round. Congruently, *Nv4*, and *Nv5*, toxin paralogs of *Nv1*, are produced in different types of gland cells in this early life stage that also differs in size [76]. These findings are supported by scRNA-seq which revealed multiple populations of glands cells to be present in this species [71].

Interestingly, there seem to be significant lineage-specific differences in venom localization in sea anemones, suggesting these systems constantly evolve. For example, the *Nv1* homolog *Av2* (also called ATX-II), which is the major neurotoxic component of the venom of the snakelocks anemone *Anemonia viridis* (also called *Anemonia sulcata* or this might be a species complex) is expressed in both ectodermal gland cells and nematocytes [69, 99]. This additional site of expression seems to be lineage specific as another species, *Anthopleura elegantissima* which is closely related to *A. viridis*, expresses its sodium channel modulator toxins only in gland cells, similarly to the distantly related *N. vectensis* [69]. Coincidently, the lineage-specific expression of *Av2* in nematocytes is correlated with a gene fusion event that resulted in the loci encoding this neurotoxin acquiring a new genomic sequence that may hold regulatory functions [100]. However, this correlation requires further investigation regarding whether this novel sequence can truly drive nematocyte-specific expression in sea anemones.

Additional examples of lineage-specific changes in the localization of venom expression in sea anemones have also been reported in a transcriptomic study [98]. Specifically, the expression of the same toxin family localized to different body regions in three different sea anemone species was reported [98]. These findings suggest that toxins might shift their expression domains along with the evolution of sea anemone species, reflecting different ecological conditions and interactions. Ultimately this modularity may allow the fast evolution of the spatial regulation of toxin expression, as each module (cell type) can more easily change its content or location across evolution, without affecting all venom components. This highlights a potential relationship between cellular complexity and the complexity of venom composition.

### Intersexual variation of the venom system

Sexual dimorphism of the venom system is another example of venom variation within a given life stage. Distinct differences in morphology or behavior between sexes of the same species are widespread among animals, including those that are venomous. Sexual dimorphism occurs through the coordination of different signals related to sex determination and differentiation. Sex determination is the primary signal guiding the embryo to develop as either male or female [101–103]. Sexual differentiation occurs following subsequent signals that further directs the primary sex-determining signal to the development of specific traits that are sexually dimorphic [104–106]. These signals can either be environmental or genetic [107–110]. The evolution and conservation of these signals are currently being resolved through comparative genomics and experimentally by using developmental genetics. Research investigating the intersexual variation of the venom system can contribute by providing robust support for the fitness implications of these traits among sexes.

#### Sexual dimorphism of the venom apparatus

Variation in the venom system among sexes has been reported across various species, including mammals, snakes, spiders, scorpions, centipedes, fish, and sea anemones (Fig. 1). One of the most striking examples of intersexual variation of the venom system is observed in the platypus (*Ornithorhynchus anatinus*). In this animal, only males inject venom through spurs that are connected to venom glands [111–113]. The function of platypus envenomation is suggested to be highly specialized, being used predominantly during mating season in aggressive encounters with other males invading territory [111–113].

A striking example of sexual dimorphism of the venom system in invertebrates is present among aculeate hymenopterans (wasps, ants, and bees). The venom system of this hymenopteran group arose from modifications of the ovipositors (the female reproductive organ which had ancestral functions related to parasitism) to become a devoted venom injection apparatus [114–116]. Subsequently, this venom system underwent functional diversification, having roles related to both predation and defense [114–116]. This highlights that venom sexual dimorphism can impact an animal’s capacity and strategy to defend against predators and capture prey.

Evidence of more subtle venom system sexual dimorphism is reported in scorpions from the genus *Centruroides*. For example, females have overall larger bodies and shorter metasoma (tail segments implicated in venom delivery), while males’ bodies are smaller their metasomal segments are larger [117, 118]. Moreover, a combination of light and transmission electron microscopy revealed that the morphometrics and morphology of male and female telsons (stinger) and venom glands differ significantly [119]. These findings highlight that male telsons are larger both cross-sectionally and volumetrically. Cell-type variation was also observed among sexes, [119] with females mostly having granule-filled cells, whereas males predominately have cells containing dissolvable vesicles. This cell type found in males is hypothesized to contribute to the observed transparent venom, characterized as “prevenom” similar to that identified by Inceoglu et al. [82]. The intersexual variation in the visual qualities of venom liquid is likely related to differences in toxin expression and venom composition.

#### Intersexual variation of toxin expression

The majority of studies investigating the sexual dimorphism of venom composition have been reported in scorpions and spiders [39, 120–127]. For example, intersexual variation in venom yield and toxicity has been observed in the Venezuelan scorpion, *Tityus nororientalis* [128]. Specifically, it was found that males have significantly higher venom yield (2.39 mg/individual) compared to female scorpions (0.98 mg/individual); however, female venom was significantly more toxic in mice. This difference in toxicity is correlated with variation in the venom composition among sexes; however, the specific toxins related to this different toxicity remain to be characterized [128].

The venom profile of the Hentz striped scorpion (*Centruroides hentzi*) revealed significant intersexual variation within and among populations [123]. Specifically, females contribute more significantly to the variation of venom between populations. In contrast, within-population venom variation is mostly driven by differences in the venom profile of males [123]. This variation within and among populations is likely contributed in part to sex-specific venom differences. This supports that selection is likely acting on the venom profile of male and female scorpions differently and contributing to the observable intraspecific variation in the venom of *C*. *hentzi* [123].

Understanding venom variation among sexes and how it relates to differences in ecological niche or courtship behavior is essential to understanding the biology of these venomous animals. Insights into this were explored in Hawaiian spiders from the genus *Tetragnatha* that utilizes different prey capture methods [122]. This comparative analysis compared adult females that spin orb webs and adult males that capture prey by wandering. In addition, other species where both sexes capture prey by wandering were also investigated [122]. Unexpectedly, differences in venom composition between males and females were observed in the species in which both capture prey by wandering [122]. This was evident with male venom composition consisting of predominately high-molecular weight components that were absent in females. In contrast, low-molecular weight components dominate the venom profile of females. The functions related to the intersexual variation of venom composition may be attributed to differences in feeding ecology or behavior as well as, mating biology, such as sexual stimulation, nuptial gifts, and/or mate recognition [122]. Further evidence of intersexual venom variation in spiders is reported for the Australian Northern (*Missulena pruinosa*) and Eastern (*Missulena bradleyi*) mouse spiders [120]. In these spiders, females from both species have a greater venom yield. Additionally, differences in prey specificity of the venom were also reported, with only the male *M. bradleyi* having vertebrate-specific toxicity. Sexual dimorphism of the venom system is also reported in venomous arthropods beyond arachnids.

The venom profile of the eastern bark centipedes (*Hemiscolopendra marginata*) exhibits significant sexual dimorphism that is driven by sex-biased gene expression [129]. This sex-biased gene expression results in males having a greater abundance of ion channel-modulating toxins, whereas γ-glutamyl transferases and CAP toxins were the most abundantly expressed components of the female venom profile. This work by Nystrom et al. [129] was the first to characterize sexual dimorphism in centipede venom and may help explain more broadly the venom variation within and among centipede species [130].

Sexual dimorphism has also been observed in venomous vertebrates, such as fish and snakes [131]. For example, this was reported in the Cano toadfish, *Thalassophryne maculosa*, which showed that among sexes, there was a difference in biochemical properties and protein abundance [132]. Concomitantly, in the Brazilian lancehead, *Bothrops moojeni*, differences in protein abundance and activity among sexes were also reported [133]. The intersexual variation of the venom system has also been described in Cnidaria, the oldest extant venomous lineage.

Among cnidarians, the intersexual variation of venom has only been reported in sea anemones*.* Specifically, it was revealed in *N*. *vectensis* that *NvePTx1* has divergent expression profiles among sexes in adults [70]. While this toxin exhibits restricted expression to the early life stages, it begins to be expressed again only in adult females localized to round structures in the mesenteries likely to be the ovaries where the eggs are formed [70, 74]. Strikingly, this sexual dimorphic expression of *NvePTx1* functions to maternally deposit this toxin into eggs during gametogenesis and sexual reproduction. The maternal deposition of toxins has ecological significance, with *N. vectensis* eggs loaded with toxins, resulting in the avoidance of killifish (*Fundulus heteroclitus*) predation [63]. Congruently, *Nv4* and *Nv5* are also loaded into the egg through maternal deposition and share similar sex-biased gene expression in female mesenteric filaments [76].

A similar pattern is observed in *Anemonia viridis* where two toxin transcripts (*Av2* and *Av7*) are found to be highly expressed in the oocyte-rich ovaries [100]. This suggests that these toxins are also maternally deposited in eggs. This is further supported by evidence of the intronless copies of *Av2* and *Av7* integrated into the genome through a process of retrotransposition [100]. The presence of these processed pseudogenes in the genome of somatic cells could only occur if the parental genes are spliced and expressed in gametes, gonads, or at an early embryonic stage [134]. Whether the maternal deposition of *Av2* and *Av7* protects the eggs of *A*. *viridis* remains to be tested. These examples of eggs being loaded with toxins can be described as transgenerational protection and provides striking evidence of how a sexual dimorphism can have a direct effect on fitness. This highlights how venom can be leveraged to understand how genotype to phenotype affects fitness. Concordantly, it allows for the direct testing of the fitness effects associated with intersexual and ontogenetic dynamics, which has critical implications in both evolutionary and developmental biology.

## Ecological significance and consequence for venom variation within and across life-history stages

Selective forces acting on the temporal separation of venom may be attributed to venom yield limitations. While venom yield varies within and among taxa, there is a finite limit to the venom load that is deliverable. For venom to effectively manipulate the target animal’s physiology, a minimum dose is required. Therefore, if venom load is finite and each component has a required proportion needed for an effect, combining all venom components into a single mixture may be disadvantageous. The separation of venom components is an elegant mechanism that would allow concentration optima while maintaining venom yield. This need to separate venom components due to limitations in venom yield may be more dramatic in earlier life stages in which venom yield is likely significantly less. For example, *N. vectensis*, in its earlier life stages relies on specific venom components for defense but also does not feed [70]. This metabolic limitation would greatly affect its ability to replenish its venom components. Furthermore, *N. vectensis* relies on both gland cells and nematocytes, with the latter cell type known to be single use [45]. Triggering and firing the stinging organelles in these cells essentially means their destruction and hence requires the production of new nematocytes and greatly increases the metabolic costs needed to replenish this defensive venom system. In these early life stages, the cost of venom production would likely be significantly higher than that in adults. Therefore, the superfluous expression of functionally unnecessary toxins could be highly detrimental to the fitness of the organism, with selection acting strongly on the temporal expression of functionally specialized toxins.

The selection in modulating toxin expression across life history may be driven by biochemical necessity. Toxins are required to be secreted following their synthesis and this demands that these peptides and proteins are soluble. Protein solubility is determined by the concentration, conformation, and quaternary structure among other factors [135–137]. Given that proteins have the capacity to convert into amyloid-like fibrils, such protein aggregation can lead to the generation of insoluble proteins leading to an inability to be secreted, as well as potentially causing cell death [137]. While aggregation can be a consequence of the overexpression of a protein, additionally, some proteins are also inherently more aggregation-prone due to biochemical properties (such as having high beta-sheet confirmation) [137, 138]. Furthermore, the overexpression of cysteine-rich peptides is also associated with enriched rates of aggregation [139]. It is hypothesized that both spontaneous intermolecular and non-specific intramolecular disulfide bond formation among proteins existing in high concentrations can lead to protein aggregation. In general, genes encoding aggregation-prone proteins are more likely to be harmful when overexpressed within a cell. This is significant as many neurotoxins found in venom are cysteine rich [2, 3, 140]. Congruently, copy number variation associated with disease and dosage-sensitive genes provides context for the need to limit the overexpression of specific genes, as an increase in gene copy number is correlated with increased protein product [141]. To avoid potential aggregation, toxin expression must be tightly regulated among populations of cells to prevent catastrophic outcomes, such as cell death. Similar evolutionary constraints have been hypothesized to drive the birth and evolution of young genes due to their enrichment in intrinsic structural disorder domains which minimize protein aggregation [142]. While this remains to be resolved [143], strong selective forces may be acting on the translational dynamics of venom components among the cells to minimize protein aggregation.

In venomous taxa, novel morphological (venom apparatus) and genetic innovations (toxin genes) co-evolve to meet the ecological requirements of an organism. Understanding the steps that lead to the evolution of a complete venom system can give important insights into the evolution and development of novelty. Intriguingly, recent findings on the evolution of the venom system in blennies have provided insights into the evolutionary steps that lead to the development of a complete venom system [144]. This work by Casewell et al. [144] revealed that enlarged canine teeth (fangs) originated at the base of the Nemophini radiation which enabled them the ability for predatory feeding. Subsequently, the evolution of deep anterior grooves and their coupling to venom secretory tissue provide *Meiacanthus* spp. with toxic venom that they effectively utilize for defense.

In addition to understanding the evolution of novelty, the trajectories that lead to complexity are also being unraveled. Comparative analysis of multiple venomous centipede species from two diverse families provided insights into the evolution of cellular and biochemical complexity of the venom system. The three species (*Thereuopoda longicornis, Scolopendra morsitans*, and *Ethmostigmus rubripes*) were all found to have low-molecular weight (< 10 kDa) toxins at varying abundances among the secretory units in the venom gland [145]. These findings support the hypothesis of previous work that the centipede venom gland is a composite of semiautonomous subglands [145, 146]. The heterogeneous toxin expression of different secretory units suggests the separation and specialization of few, highly abundant venom components among subglands. The study by Undheim et al. [145] also revealed that the diversity of venom composition correlates with the venom gland’s cellular complexity [145]. Specifically, the *T*. *longicornis* gland contains ∼1,000 individual secretory units, compared to the venom glands of *S. morsitans* and *E. rubripes* which contain 10- to 100-fold more secretory units. The venoms from *S. morsitans* and *E. rubripes* are also observed to be much more complex [145]. Potentially, the evolution of venom complexity may be driven by the combinatorial expression of toxin genes between diverse secretory units. It is plausible that the evolution of greater gland complexity through the amplification of secretory units facilitated the biochemical diversification of the centipede venom arsenal. Furthermore, individual peptide masses identified as toxins appear to be localized to distinct regions along the length of the venom gland. This observation is strikingly similar to that reported in cone snails [83]. However, their capacity to compartmentalize venom components related to predation and defense remains to be tested.

The distribution and evolution of intersexual variation of venom is currently highly patchy among venomous lineages. While this may be due to the limited number of studies investigating this phenomenon, further work is therefore required to see if this is in fact common among venomous animals. From an evo-devo perspective, this suggests that these animals would have convergently evolved gene regulatory networks capable of separating venom among sexes, and whether these were recurrently co-opted from the same network might provide insights more broadly into the mechanisms that underlie convergent evolution of venom. The function of intersexual venom variation is largely attributed to having functions related to mating behavior and sexual stimulation, nuptial gifts, and/or mate recognition and aggression among conspecifics [39, 120–127]. Progeny protection is another interesting function related to intersexual venom variation, such as eggs being loaded with venom from the mother, or males having a higher protein yield and potency for guarding eggs against predators and conspecifics. Furthermore, theoretical and empirical evidence suggests that males and females should be under selection for different dietary preferences and resource utilization that maximize their sex-specific fitness [147, 148]. This can be explained by evidence that different dietary requirements are needed to maximize their fertility may not be the same. For example, in fruit flies, females benefit most from foods that contain lots of protein, while males are more fertile when they eat foods that are rich in carbohydrates [149, 150]. The function of venom in many animals is related to prey capture which would directly affect the animal’s diet. Differences in prey preferences leading to sex-specific dietary requirements may explain the intersexual venom variation observed in some lineages.

## Future prospects and concluding remarks

Evolutionary developmental biology is a field that utilizes comparative biology approaches in order to understand the evolution of developmental processes [151, 152]. The recent link between developmental processes and venom dynamics brings together venom research and evo-devo. Furthermore, the comparative biology approaches that are at the very core of evo-devo as a discipline are also highly relevant for the studies of how venom systems and venom dynamics evolve. Thus, these two fields, which until only a few years ago seemed to be completely detached from one another, are coming closer to one another and we propose that venom research, especially in evolutionary terms, can gain much from adopting the practices and mindset that typify evo-devo.

We believe that another shared feature that brings these two fields together is the “omics” (genomics, transcriptomics, and proteomics) revolution that strongly affected all of the biological disciplines, but truly transformed both evo-devo and venom research (especially the sub-discipline called “venomics” see review Sunagar et al. [153]) and brought them closer to one another from a technological point of view. A major commonality between the fields that made “omics” so valuable for them is the focus on non-model organisms. The ability to use “omics” enabled studying those organisms that were difficult to study due to technological limitations. Indeed, a major bottleneck in the study of venom systems via an evo-devo perspective is the quite restricted accessibility of many venomous animals in the early developmental stages of their lives as well as the very limited toolbox available for the genetic manipulation of venomous animals. In this respect, several cnidarian species are an outlier for more than a decade [154–160]. However, other venomous species, such as the house spider or parasitic wasps, become amenable for genetic manipulation, expanding the possibility of studying the developmental evolution of venom systems in non-cnidarian species [161–164]. Moreover, the genetic manipulation revolution of the last several years where new techniques based on the CRISPR/Cas9 system enable the genetic engineering of essentially any eukaryotic organism that can be grown or obtained as a zygote could revolutionize this neglected aspect of studying venom systems evolution [154–157, 159, 161, 165]. One noticeable example is Cas9-based mutagenesis of the honeybee, *Apis mellifera* [165]*,* arguably the venomous animal which holds the greatest economical and agricultural importance. Even when such advanced genetic tools remain unavailable for many venomous species, the ability to compare venom systems at the morphological, biochemical, and genetic levels can be highly informative for understanding this evolutionary innovation in different lineages. Altogether, we believe that the fusion of venom research with the comparative frame of mind of evo-devo results in an exciting development that can teach us about the important aspects of venom evolution in a novel perspective that is lacking from a field that traditionally focused on pharmacological and even translational aspects and less on evolution or the temporal dimension that can hide significant and fascinating biological complexities.

## Data Availability

Not applicable.

## Abbreviations

AG: Accessory gland
ASC: Adult stem cells
AMG: Anterior main gland
CRISP: Cysteine-rich secretory protein
EGF: Epidermal growth factor
FGF10: Fibroblast growth factor 10
HPF: Hours post-fertilization
i-cells: Interstitial cells
MSI: Mass spectrometry imaging
PMG: Posterior main gland
K+: Potassium
PGE2: Prostaglandin E2
SHH: Sonic hedgehog
TGF: Transforming growth factor
SCRiPs: Small cysteine-rich peptides
